# Population health and community health: brokering the two through art and community engagement

**DOI:** 10.3389/fpubh.2024.1480795

**Published:** 2024-11-28

**Authors:** Panagis Galiatsatos, Kimberly Hailey-Fair, Marcie Johnson, Elisabeth A. C. Vanderpool, Rosalyn W. Stewart, Karen Alexander, Susan Magsamen

**Affiliations:** ^1^Division of Pulmonary and Critical Care Medicine, Johns Hopkins School of Medicine, Baltimore, MD, United States; ^2^Office of Diversity, Inclusion, and Health Equity, Johns Hopkins Health System, Baltimore, MD, United States; ^3^Medicine for the Greater Good, Johns Hopkins School of Medicine, Baltimore, MD, United States; ^4^Department of Pediatrics, The Johns Hopkins University School of Medicine, Baltimore, MD, United States; ^5^International Arts + Mind Labs, The Johns Hopkins University School of Medicine, Baltimore, MD, United States

**Keywords:** community engagement, arts, homophily, health equity, population health, community health

## Abstract

The arts and aesthetic experiences have fostered and enhanced relationships between diverse, distinct groups in an effort to build comradery, trust, and engagement. In regards to collaborations between health systems and communities, taking into account strategies to build such relationships is vital in an effort to assure a bidirectional collaboration that implements public health insight and resources effectively. There are many factors warranting consideration when building effective community engagement for health promotion between healthcare systems and local community organizations and residents. Such factors include, but are not limited to, homophily, transitivity, structural holes, and maintaining weak ties. In this brief review, we will explore how the arts can be utilized to broker relationships for healthcare systems implementing community engagement with partnering, diverse social networks. Specifically, we will explore the role of the arts and aesthetic experience to create homophily, foster transitivity and balance, enhance collaboration and build meaningful connections between healthcare systems and social networks to more effectively address health concerns for all involved.

## Introduction

The ancient Greeks of Massalia, France, arrived between 500 and 300 BCE from Phocaea (western modern day Turkey) (1). Given two diverse cultures from two ends of the Mediterranean coming together, what appears to have allowed the establishment and acceptance of both communities was a wedding, as told in the founding myth of Marseille by Aristotle (1). The wedding was filled with cultural diets and festivities, bridging these two communities and solidifying them. The western Mediterranean is filled with many similar stories of cities meeting the arrival of eastern Mediterranean sailors who brought their culture and the arts as a way to establish engagement, trust, and, at the end, settlement (2). And such findings continue to be present in over 5,000 indigenous tribes that still exist throughout the world, making arts as an everyday ritual and practice is essential. While the word “art” didn't exist in early cultures, acts of creative expression were totally inseparable from daily living (3). They were integrated in the everyday, and the urge to practice creative acts was universal (3). Art creates culture, culture creates community and community creates humanity.

Throughout the millennia, the arts and aesthetic experiences have fostered and enhanced relationships between diverse, distinct groups. For example, in modern times, to cultivate diplomacy between two countries, it is common to disseminate one country's arts, be it in music, theater, or museums, across nations (4). The United States has arts diplomacy programs in the State Department with the purpose of sharing both country specific and universal themes to unite diverse communities. And for other populations, art is used to reaffirm their culture and promote social cohesiveness. For example, for the Inuit and Metis populations of Canada, the practice of art in their healing programs promotes self-development, cultural safety, and improved social relations, even in the setting of competing contextual challenges from other cultural and national intrusions; thereby, any connectivity to such a population would warrant an understand of how their art reaffirms their respective identity and cultural priorities (5, 6). There are also programs around the world geared to peace-making, conflict resolution and complex decision making at government, business and community levels—all through the lens of the arts, as neuromodulators for transformation. We are the only species that has evolved to creatively express ourselves through the language arts and aesthetics. There's a humanity in the arts that results in a shared sense of awe, wonder, enjoyment and understanding, regardless of the culture, customs or language Further, the arts itself may serve as a catalyst to foster togetherness between distinct groups and, ultimately, trust for the purpose of collaborations and relationships. And, through the emerging interdisciplinary field of neuroaesthetics, the study of how the arts and aesthetic experiences measurably change our brains, bodies and behaviors and how this knowledge can be translated into solutions in health, wellbeing, learning, and community development, we now know that we are neurobiologically and physiologically wired for the arts.

In today's world, healthcare institutions are abandoning traditional approaches to care and investing in novel strategies that embrace institutional outreach and community collaboration to achieve significantly better outcomes for patients, families and healthcare practitioners. Innovative health promotion initiatives are occurring in non-traditional setting that include, but are not limited to, faith-based organizations, schools, and barbershops (7–10). This approach to healthcare was reaffirmed during the early years of the COVID-19 pandemic, where messaging COVID-19 mitigation strategies and vaccinations were effective if and when messengers went into local communities (11–13). In the context of COVID-19, the population health goal of healthcare systems was to reduce infections and hospitalizations; to do this successfully, the healthcare systems had to understand community health issues that would attenuate mitigation strategies and/or vaccine acceptance. Such an understanding for all health matters, would be most effective if community engagement is used to bridge population health strategies and community health goals (14).

There are many factors warranting consideration when building effective community engagement for health promotion between healthcare systems and local community organizations and residents. Such factors include, but are not limited to, homophily, transitivity, structural holes, and maintaining weak ties. In addition, trust is a key factor valuable for both internal participants of a social network and between the healthcare system and community organization. Change happens at the speed of trust and in communities that have experienced harm from medical and academic institutions there are often reparations and healing that must occur.

In this brief review, we will explore how the arts can be utilized to broker relationships for healthcare systems implementing community engagement with partnering, diverse social networks. Specifically, we will explore the role of the arts and aesthetic experience to create homophily, foster transitivity and balance, enhance collaboration and build meaningful connections between healthcare systems and social networks to more effectively address population health objectives and community health priorities.

## Creating homophily

Homophily refers to the tendency for persons to interact with one another due to others having characteristics that resemble them (15). Such a variable is often key to create (and sustain) social networks that allow for efficient interactions, engagements, and exchange of information and resources (16). Further, more characteristics shared between persons within a social network often result in a greater social cohesiveness, which in of itself results in more trust and fostering a sense of inclusivity for common interests and goals among all members (17, 18).

There are two types of homophily. Status-homophily can be ascribed (age, race, ethnicity, and sex) or acquired (education, occupation, and hobbies), while value-homophily includes shared attitudes, stereotypes, and opinions (19). Therefore, some factors of homophily can be modified and/or evolve. Causes of homophily may be linked to the recognition that persons with common norms, values, and attitudes often come together with persons with common attributes. In addition, the reverse is true, in that persons with common attributes over time may develop the same common norms, values, and attitudes if they are together often (19). Another cause of homophily is often due to structural location. Persons coming together to operate in the same space may have some similar attributes that can result in relationships and shared norms (19). The latter is often seen, for example, in classroom settings, where students gather for a topic, and over time, find commonality with one another and build relationships. This specific development of homophily is one way art can be utilized for community engagement. The arts bring people together in a shared neural space, for a shared experience, fostering togetherness and commonality. It also offers an opportunity to share diverse and shared beliefs, values and ideas, enhancing empathy through perspective-taking.

There is a positive effect of cultural similarity on social networks and their respective ties (20). Specifically, upon drawing on cultural similarities in emerging groups and relationships, cultural homophily may stimulate and reinforce social ties, such as weak ties, between different organizations and groups, contesting objective relations in addition to reproducing them (20). The arts are a significant cultural variable that finds common ground in many individuals, even in those with varying ascribed factors of homophily. Therefore, using the arts, in many modalities, as a collective activity to broker relationships between two diverse organizations may assist in achieving three goals: (1) collaborate on a unified project and (2) sustain the collaboration with a relationship that includes some degree of trust (3) understand the other through story and narrative. For example, a health system may attend a social network's worship service (the social network being a faith-based organization) and participate in choir singing of religious hymns. Thereby, through music and its participation, one social network (the faith-based organization) sees another engaging in culturally significant activities, while the other (the health system) learns more about the potential collaborating party through the music. Additionally, neuroscience is proving that when we sing together, we entrain and synchronize with the music, thus creating new neural connections (21–23).

## Improving transitivity and balance

Transitivity describes the property of the relationships between persons and organizations (24). While there may be different combinations of relationships between persons within organizations, how these transitive relationships take place will play a role in how information, ideas, and resources flow from one person to another. Further, balance within organizational and between organizational relationships is critical. In some relationships with a formal hierarchy, the balance is uneven in regards to the direction of information and ideas. However, in other relationships, especially ones in which two diverse organizations attempt to build a collaboration, the transitivity of the relationship should be built on the balance of a mediator, reaffirming the aphorism of “a friend of my friend is a friend of mine.”

The arts and aesthetic experiences can be the tools that help to foster such a desired balance and improves the transitivity of a relationship. Having a healthcare system and a community organization come together in some capacity where the objective is art making or beholding, such as a live musical band or engagement in a story-telling results in both parties can dissolve formal hierarchies by participating in events that recognize all persons involved on equal footing. Further, such events improve the relationships between two seemingly distinct groups in that they may result in a new factor of commonality (and, thereby, improve homophily).

The goal of recognizing these social elements of transitivity and balance are meant to foster closer relationships between health systems and the communities with which they engage. It is undoubtedly easier to implement ideas, share information, and distribute resources when there is a bidirectional flow between both parties, the relationships are balanced, and a significant transitive state exists.

## Maintaining weak ties

Weak ties are defined as connections between otherwise-distant parts of a social network, joining in order to overcome structural holes and gaps within that network (25). For example, a faith-based organization finding themselves having a surge of congregants struggling with diabetes may reach out to a health system for education and information. In addition, a health system may find themselves with a surge of patients hospitalized with diabetes, unsure of the social factors that are attenuating the outpatient management. Therefore, the coming together of these social networks due to their respective structural gaps creates a weak tie.

However, once the engagements end for a specific project, how does the relationship change in an effort to maintain the connection? This, again, is where the arts and aesthetic experiences can play an important community development role. The arts can help to maintain and deepen relationships and their respective weak connections, increasing understanding and trust, especially during times without active engagements or targeted projects.

For instance, a visual artist might connect with a local community organization to create public art installations that address social issues such as diabetes awareness. This collaboration can bring diverse perspectives and skills, bridging gaps between different sectors of the community and enriching both the artistic and social impact. Poets and spoken word artists could contribute by using their craft to articulate the nuanced emotions and experiences associated with illness and healing. Poetry readings and workshops can be held in hospitals, clinics, and community centers, providing a therapeutic outlet for patients and caregivers alike. Poets can also collaborate with medical professionals to create written works that communicate complex health information in a more accessible and emotionally resonant manner. By giving voice to the often-unspoken aspects of the medical experience, poets help to bridge the gap between clinical and personal perspectives, fostering stronger connections within the community.

Musicians could utilize their music to address and bring awareness to health issues, thereby connecting diverse groups. For instance, benefit concerts or musical events can be organized to raise awareness about diseases such as diabetes or mental health conditions. These events can draw in different segments of the population, including healthcare professionals, patients, and community members, creating a shared space for dialogue and support.

Artists from a variety of disciplines could collaborate on multidisciplinary projects that address health issues from multiple angles. For example, a community health campaign might include a combination of musical performances, theatrical productions, and poetry readings, all centered around a common theme such as chronic illness management or mental health awareness. These collaborative efforts can engage a broader audience and create a more cohesive network of support and education. By nurturing these connections, art communities can expand their reach and influence, enhancing both cultural dialogue and community wellbeing.

Fostering relationships, nurturing connection takes time. It is essential to fundamentally addressing vital public health issues that almost always require strong bi-directional relationships and active partnerships (e.g., during the COVID-19 early months of the pandemic). Therefore, creating moments where the social networks can come together through the arts and aesthetic experiences (e.g., health fairs, musical events) is an investment in community health and will build and maintain such ties, creating a sustainable network in of itself.

## Next steps

The applicability of the arts into community engagement should come with accountable outcomes for all parties involved, knowing these outcomes may take time to find an appropriate timeline. Broad knowledge, flexibility of thought, openness to unanticipated connections, along with an immersion of a flow of ideas are all critical to effective community engagement, and probable outcomes when the arts are used as a tool for such engagement. However, objectifying the outcomes will be vital, to reaffirm the arts' role and to create a sense of accountability that should accompany these collaborations. Objective analyses of these social networks focused on end outcomes (e.g., behavioral change, such as health factors on improved blood pressure management to smoking cessation) should be met with surrogate outcomes as well (e.g., meetings, feedback given and acted upon, and leadership and staff consistency). Therefore, collaborations should set forth testing of specific goals and outcomes, and highlight how the arts assist in facilitating these achievements.

## Conclusion

As community engagement continues to grow as a valuable tool for implementing population health strategies for health systems and for achieving community health goals in communities, all parties must recognize that the social elements that strengthen the engagements must be prioritized. These social elements assure trust and a frictionless flow of resources and efforts to achieve the health goals set forward. The arts and aesthetic experiences should be seen as essential tools to achieve homophily, balance, and transitivity, social elements that are responsible for the success of creating and maintaining ties between distinct social networks. At the same time, these collaborations should identify targets to achieve, targets that the arts either directly result in or influence, and that reaffirm the optimal health impact of a community engagement effort and social networking partnerships. If medicine is to be a public trust, it must emphasize valued aspects of public life in which communities heal, grow and thrive through culture and the arts –amplifying the humanity in all.
